# Evaluation of the free-radical scavenging and antioxidant activities of Chilauni, *Schima wallichii* Korth *in vitro*

**DOI:** 10.4155/fsoa-2017-0086

**Published:** 2018-01-04

**Authors:** K Lalhminghlui, Ganesh Chandra Jagetia

**Affiliations:** 1 Department of Zoology, Mizoram University, Tanhril, Aizawl-796 004 Mizoram, India

**Keywords:** antioxidant, DPPH, flavonoid, hydroxyl, *Schima wallichii*

## Abstract

**Aim:**

Free radicals are an outcome of various metabolic activities and their excess production leads to many diseases. Therefore, it is necessary to neutralize excess free radicals.

**Materials & methods:**

Free-radical scavenging activity of various extracts of *Schima wallichii* was evaluated using standard protocols.

**Results:**

Chloroform, ethanol and aqueous extracts of *S. wallichii* scavenged DPPH, hydroxyl, superoxide, nitric oxide and ABTS free radicals and increased ferric-reducing antioxidant potential in a concentration-dependent manner. A total of 1000 μg/ml of all the extracts and ethanol extract showed highest total flavonoids and phenol contents, respectively.

**Conclusion:**

The different extracts of *S. wallichii* scavenged different free radicals efficiently due to the presence of flavonoids and polyphenols and may be helpful in free radical-induced diseases.

## Lay abstract

Free radicals induce several health disorders including cancer, and their excess generation needs to be controlled. Our study demonstrates that *Schima wallichii* scavenged DPPH, hydroxyl, superoxide, nitric oxide, ABTS free radicals and increased ferric-reducing antioxidant potential in a concentration-dependent manner. It could be one of the agents able to mitigate excess free-radical generation and ward off free radical-induced inflammation and diseases.

## Graphical abstract

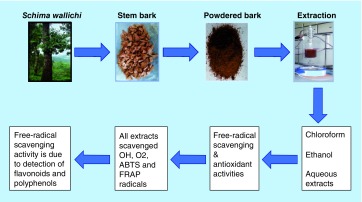

Usage of traditional medicines and other medicinal plants as therapeutic agents for maintaining proper health has been practiced widely in developing countries [1]. Plants and other natural products are still in great demand due to various factors like their safety, dependability and lesser side effects [2]. The greater adverse side effects caused by many cancer chemotherapeutic drugs may have been the main driving force to the use of alternative medicine in the hope of a better cancer cure. Approximately 80% of the world's inhabitants rely mainly on traditional medicines for their primary healthcare indicating that plant-based, traditional medicine systems will continue to play a major role in human healthcare in the future [3–5]. The interest in medicinal plants in healthcare has been rekindled recently due to the rising costs of prescription drugs for maintaining the proper health of an individual and their well being. The bioprospecting of new drugs derived from plants could be more economical with lesser side effects or no toxicity at all [6].

Free radicals are molecules or fragments of molecules that contain an unpaired electron in their atomic or molecular orbitals or simply reactive oxygen species, which in addition also contain other oxygen species including hydrogen peroxide that are highly reactive moieties and are generated by cells during respiration, and cell-mediated immune functions [7,8]. They are produced naturally in the body as they play an important role in many cellular functions. However, their high production induces molecular and cellular damage leading to the development of various human health disorders including cancer [9,10]. The excess free radicals produced during respiration and other activities could cause various damages leading to loss of function and eventually death of the organism [11]. Reactive oxygen species-induced damage can be alleviated using certain substances known as antioxidants, which are molecules capable of inhibiting oxidation of other molecules. The antioxidants are helpful in reducing and preventing damage from free-radical reactions because of their ability to donate electrons that can neutralize the radical formation [12–14]. Many plants synthesize secondary metabolites naturally, including flavonoids and polyphenols which act as antioxidants and also play a critical role in different biological activities [15–17]. Therefore, plants and natural products could be a major source of antioxidants that can scavenge free radicals and protect from excess oxidative stress-induced ailments.

*Schima wallichii* (DC) Korth, Chilauni or the needle wood tree, is an Asian species of evergreen tree belonging to the tea family, Theaceae. The genus inhabits warm temperate to subtropical climates across southern and Southeast Asia, ranging from the Eastern Himalaya of Nepal to eastern India across Indochina, southern China, Taiwan and the Ryukyu Islands. It usually grows up to 35 m in height and in some places, it may be 40 feet tall [18]. Locally, it is called ‘khiang’ in the Mizo language. *S. wallichii* is known to possess several medicinal properties. Traditionally, the leaves and the stem bark are normally used. The bark is used as an antiseptic for cuts and wounds, and as a cure for gonorrhea. It acts as a vermicide and a skin irritant [19]. Decoction of bark is good for fever and is effective against head lice infection [20]. The bark juice of Chilauni is used in animals as a liver fluke disinfesting agent [21]. The sap from its stem is used for curing ear infection [22]. Fruit juice of Chilauni is used by the people of western Mizoram, India against snakebite [21,23]. Its young plants, leaves and roots are also used medicinally against fever. The bark of *S. wallichii* is anthelmintic and rubefacient [24]. The leaves of *S. wallichii* are known to have antitumor and antimutagenic properties [25,26]. Kaempferol-3-rhamnoside, a compound isolated from the leaves of *S. wallichii* inhibited MCF-7 breast cancer cell proliferation through activation of the caspase cascade pathway [27].

Cancer cells are always at elevated oxidative stress, which offers a survival advantage to them, therefore we reasoned that if *S. wallichii*, which is used ethnomedicinally in traditional systems to treat various disorders would possess antioxidant potential, could be useful as an anticancer agent. Keeping this in mind we have evaluated the free-radical scavenging activity of various extracts of *S. wallichii in vitro*.

## Materials & methods

### Chemicals & reagents

Analytical grade chemicals and Milli Q water were used for the entire analyses. Ascorbic acid, 2,2′-azino-bis(3-ethylbenzothiazoline-6-sulfonic acid) diammonium salt (ABTS), dimethyl sulfoxide (DMSO), 1,1-diphenyl-2-picrylhydrazyl (DPPH), ethylenediaminetetraacetic acid (EDTA), β-nicotinamide adenine dinucleotide (NADH), 2,4,6-tris(2-pyridyl)-s-triazine (TPTZ), nitroblue tetrazolium (NBT), phenazine methosulfate (PMS), trichloroacetic acid (TCA), sodium nitroprusside and (N-(1-naphthyl)ethylenediamine dihydrochloride  (NED or Griess reagent) were supplied by Sigma-Aldrich Chemical Co (Bangalore, India). Aluminum chloride, ethanol, methanol, ferric chloride, Folin-Ciocalteu reagent, potassium chloride, sodium acetate, sodium carbonate, sodium hydroxide, sodium chloride, disodium hydrogen phosphate (anhydrous), potassium dihydrogen phosphate, potassium acetate, gallic acid, ferrous ammonium sulfate, ammonium acetate, glacial acetic acid and acetyl acetone were requisitioned from Merck (Mumbai, India).

### Preparation of extracts

*S. wallichii* (family: Theaceae) was identified by the Department of Horticulture Aromatic and Medicinal Plants, Mizoram University, Aizawl, India and authenticated by the Botanical Survey of India, Shillong (BSI/ERC/Tech//Identification/2017/570). The noninfected and matured stem bark of *S. wallichii* was collected from Bazar Veng, Lunglei, Mizoram, India during the months of April and May. The bark was cleaned and shade dried at room temperature in clean and hygienic conditions. The dried bark was powdered using an electrical grinder and was extracted sequentially with petroleum ether, chloroform, ethanol and distilled water according to increasing polarity using a Soxhlet apparatus. The liquid extracts were filtered and concentrated by evaporating them to dryness under reduced pressure. The concentrated extracts were stored at -80°C until use.

### Experimental protocol

The free-radical scavenging activity of different extracts of *S. wallichii* was estimated according to standard protocols as described below.

#### DPPH free-radical scavenging assay

The DPPH free-radical scavenging activity of *S. wallichii* was estimated as described earlier [28]. Various concentrations of different extracts of *S. wallichii* (0.5 ml each) were mixed thoroughly with 1-ml methanol solution of 0.1 mM DPPH. The mixture was allowed to stand for 30 min in the dark. The absorbance was measured at 523 nm using a UV/VIS Spectrophotometer (Eppendorf India Limited, Kolkata, India). An equal amount of DPPH and methanol were used as standard and blank, respectively. The scavenging activity was calculated using the following formula:

Scavenging (%) = (A_control_ - A_sample_)/A_control_ × 100,

where A_sample_ is the absorbance of the test sample and A_control_ is the absorbance of the control.

#### Hydroxyl radical scavenging assay

The hydroxyl radical scavenging activity of *S. wallichii* was assayed according to the earlier described method [29] with minor modifications. The reaction mixture contained deoxyribose (2.8 mM), KH_2_PO_4_-NaOH buffer, pH 7.4 (0.05 M), FeCl_3_ (0.1 mM), EDTA (0.1 mM), H_2_O_2_ (1 mM) and different concentrations of *S. wallichii* extracts in a final volume of 2 ml. The mixture was incubated at 37°C for 30 min followed by the addition of 2 ml of trichloroacetic acid (2.8% w/v) and thiobarbituric acid. Thereafter it was kept for 30 min in a boiling water bath, and cooled. The absorbance was recorded at 532 nm in a UV–VIS spectrophotometer. Gallic acid was used as the standard and the results have been expressed as gallic acid equivalent.

#### Superoxide anion scavenging assay

Scavenging of the superoxide (O_2_^•-^) anion radical was measured using a modified method [30]. The reaction mixture contained 0.2 ml of NBT (1 mg/ml of solution in DMSO), 0.6 ml different extracts, 2 ml of alkaline DMSO (1 ml DMSO containing 5 mM NaOH in 0.1 ml H_2_O) in a final volume of 2.8 ml. The absorbance was recorded at 560 nm using a UV–VIS spectrophotometer. The blank consisted of pure DMSO instead of alkaline DMSO. The results have been expressed as ascorbic acid equivalent which was used as a standard.

#### ABTS scavenging assay

ABTS scavenging activity of different extracts of *S. wallichii* was determined as described earlier [31]. Briefly, 37.5 mg of potassium persulfate was dissolved in 1 ml of distilled water. A total of 44 μl of this solution was added to 9.7 mg of ABTS dissolved in 2.5 ml of distilled water so as to prepare ABTS solution. The ABTS solution was allowed to stand in the dark for about 15 h at room temperature. The working solution was prepared by mixing 1 ml of ABTS solution with 88 ml of 50% ethanol. A total of 25 μl of different concentrations of chloroform, ethanol or aqueous extract of *S. wallichii* were mixed with 250 μl of ABTS working solution and allowed to stand for 4 min. The absorbance was read at 734 nm in a UV–VIS spectrophotometer. The results have been expressed as ascorbic acid equivalent which was used as a standard.

#### Nitric oxide scavenging assay

The nitric oxide scavenging activity was estimated according to the earlier described method [32]. Sodium nitroprusside (5 mM) in phosphate buffered saline was mixed with different concentrations of the chloroform, ethanol or aqueous extract of *S. wallichii* and incubated at 25°C for 150 min. The samples were then mixed with Griess reagent (1% sulfanilamide, 2% H_3_PO_4_ and 0.1% N-(1-naphthyl)ethylenediamine dihydrochloride). The absorbance of the chromophore formed during diazotization of nitrite with sulfanilamide and subsequent coupling with NED was read at 546 nm using a UV–VIS spectrophotometer. The inhibition of nitric oxide formation was determined with respect to standard potassium nitrite in the same way with Griess reagent. The results have been expressed as potassium nitrite equivalent which has been used as a standard.

#### Ferric-reducing antioxidant potential assay

The ability of different *S. wallichii* extracts to decrease ferric ion production was measured as described earlier [33] with minor modifications. A total of 50 μl of various concentrations of chloroform, ethanol or aqueous extract were added to 3 ml of ferric-reducing antioxidant potential (FRAP) reagent (ten parts of 300 mM acetate buffer, pH 3.6, one part of TPTZ solution and one part of 20 mM Fecl_3_.6H_2_O solution) and the reaction mixture was incubated at 37°C for 30 min. The increase in absorbance was measured at 593 nm using UV–VIS spectrophotometer. The antioxidant activity of the extracts is based on their ability to reduce ferric ions and it has been expressed as milligram ferrous sulfate equivalents/100 g of *S. wallichii* extracts.

#### Determination of total phenolic contents

The total phenolic contents of the *S. wallichii* extracts were determined as described earlier [34]. Briefly, 500 μl of different extracts of *S. wallichii* were mixed with 1000 μl of 1:10 Folin-Ciocalteu's reagent and incubated at room temperature for 5 min followed by the addition of 900 μl saturated (7.5%) sodium carbonate solution. After 1 h of incubation at room temperature, the absorbance was recorded at 765 nm using UV–VIS spectrophotometer. The total phenolic contents of the extracts have been expressed as gallic acid equivalents mg/100 g of the extracts.

#### Total flavonoids determination

The total flavonoids were determined by colorimetric method described earlier [35]. 1 ml of chloroform, ethanol or aqueous extract of *S. wallichii* was individually mixed with 1.5 ml of 95% methanol, 0.1 ml of 10% aluminium chloride, 0.1 ml of 1 M potassium acetate and 2.8 ml of distilled water and thereafter incubated for 30 min at room temperature. The absorbance of the reaction mixture was recorded at 415 nm with a UV–VIS spectrophotometer. The presence of flavonoids in *S. wallichii* extracts was expressed as milligram quercetin equivalent/100 g of the extract/s.

## Results

**Figure 1.**
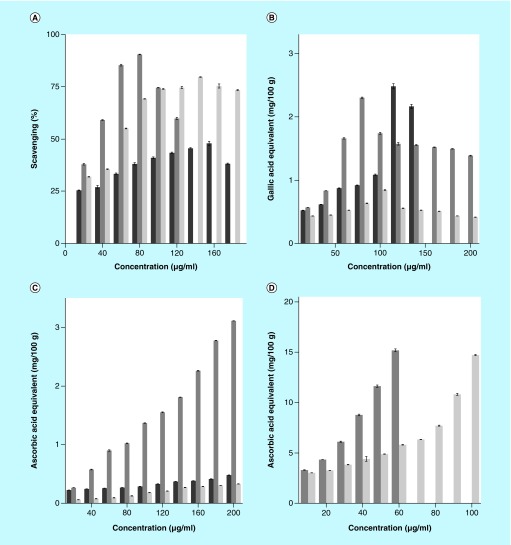
**The free-radical scavenging activity of different stem extracts of *Schima wallichii*.** **(A)** DPPH, **(B)** hydroxyl, **(C)** superoxide and **(D)** ABTS radicals. Dark gray: chloroform extract; gray: ethanol extract; and light gray: aqueous extract. The data are expressed as mean ± standard error of the mean; n = 5.

**Figure 2.**
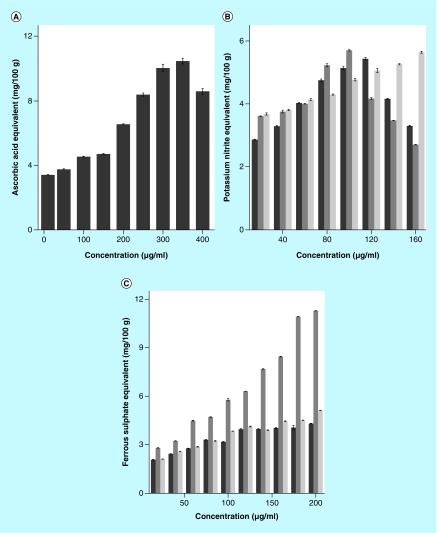
**The free-radical scavenging activity of different stem extracts of *Schima wallichii*.** **(A)** ABTS (CHCl_3_), **(B)** Nitric oxide, **(C)** Ferric-reducing antioxidant potential (FRAP) radicals. Dark gray: chloroform extract; gray: ethanol extract; and light gray: aqueous extract. Values are expressed as mean ± standard error of the mean; n = 5.

**Figure 3.**
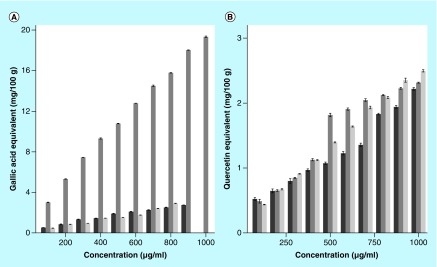
**The total phenol and flavonoid contents of different extracts of *Schima wallichii* (100–1000 μg/ml).** The data are expressed as mean ± standard error of the mean; n = 5. **(A)** Total phenols and **(B)** flavonoids.

The results of free-radical scavenging by different extracts of *S. wallichii* are shown as mean ± standard error of the mean in Figures 1 and 2, whereas that of total phenols and flavonoids in Figure 3.

### DPPH free-radical scavenging

The chloroform, ethanol and aqueous extracts of *S. wallichii* showed a concentration-dependent rise in the scavenging of DPPH free radicals and a maximum scavenging activity was recorded at a concentration of 160, 80 and 140 μg/ml chloroform, ethanol and aqueous extracts, respectively. Thereafter, the scavenging effect declined (Figure 1). The ethanol extract was best as its low concentration scavenged higher amount of DPPH free radicals (Figure 1).

### Hydroxyl radical scavenging

The scavenging of hydroxyl radicals depended on the dose of extracts of *S. wallichii.* The chloroform, ethanol and aqueous extracts inhibited the generation of hydroxyl radicals in a concentration-dependent manner and a maximum inhibition in ^•^OH generation was observed at 80 μg/ml for ethanol, 100 μg/ml for aqueous and 120 μg/ml for chloroform extracts (Figure 1).

### Superoxide anion scavenging

The chloroform, ethanol and aqueous extracts of *S. wallichii* showed a concentration-dependent increase in the inhibition of superoxide generation and the highest scavenging activity for O_2_^•-^, was observed at a concentration of 200 μg/ml for all the three extracts (Figure 1).

### ABTS scavenging

Various extracts of *S. wallichii* showed a concentration-dependent rise in the scavenging of the ABTS free radicals (Figures 1 & 2). The maximum activity for chloroform extract was recorded for 350 μg/ml (Figure 2), whereas ethanol and aqueous extracts showed maximum ABTS inhibitory action at 60 and 100 μg/ml, respectively (Figure 1). The ethanol extract proved to be the best among all the three extracts as it has maximum effect at a lower concentration (Figures 1 & 2).

### Nitric oxide scavenging

The analysis of nitric oxide scavenging activity also revealed a concentration-dependent rise in its scavenging by chloroform, ethanol and aqueous extracts of *S. wallichii* (Figure 2). The greatest scavenging activity was discernible at 120, 100 and 160 μg/ml for chloroform, ethanol and aqueous extracts, respectively, which declined thereafter (Figure 2).

### Ferric-reducing antioxidant potential

The FRAP  of chloroform, ethanol and aqueous extracts of *S. wallichii* showed a concentration-dependent rise up to 200 μg/ml, the highest concentration evaluated. All extracts were equally effective in scavenging the FRAP radical (Figure 2).

### Determination of total phenolic contents

Total phenol contents of *S. wallichii* extracts showed a concentration-dependent rise up to a concentration of 900 μg/ml for chloroform, 1000 μg/ml for ethanol and 800 μg/ml for aqueous extracts (Figure 3).

### Total flavonoids contents

The chloroform, ethanol and aqueous extracts of *S. wallichii* showed a concentration-dependent increase in the total flavonoid contents. The maximum quantity of flavonoids was estimated for 1000 μg/ml for chloroform, ethanol and aqueous extracts, respectively (Figure 3).

## Discussion

Free radicals are closely associated with oxidative damage and antioxidants are reducing agents, which limit oxidative damage to biological structures by donating electrons to free radicals and passivating them [36]. The interaction of oxygen with certain molecules leads to the formation of free radicals and once formed, the chief danger comes from the damage they can inflict when they react with important cellular components including DNA, proteins and the cell membrane [37]. These free radicals interact with the antioxidants, which can eventually neutralize them before damages are initiated [38]. Plants synthesize several compounds as secondary metabolites and many of them act as antioxidants. Therefore, the present study was undertaken to study the free-radical scavenging ability of *S. wallichii in vitro*.

DPPH is a dark-colored crystalline powder composed of stable free-radical molecules. Most notably, it is a common antioxidant assay and is a well-known radical. DPPH radical has a deep violet color in solution, and it becomes colorless or pale yellow when neutralized and converted into DPPH-H [39]. Many plant extracts have been reported to scavenge DPPH radicals *in vitro* [2,21,40–43]. Different extracts of *S. wallichii* scavenged DPPH radicals in a concentration-dependent manner. Similarly, different tea extracts containing a number of polyphenols have been reported to scavenge DPPH free radicals [44]. Kaempferol present in several plants including *S. wallichii* has been reported to scavenge DPPH free radicals earlier with an IC50 value of 0.004349 mg·ml^-1^ [45–47]. Other phytochemicals like mangiferin and naringin have been reported to scavenge DPPH radicals in a concentration-dependent manner [40,48]. The scavenging activity for ethanol extracts of *S. wallichii* was 80 and 140 μg/ml for aqueous extracts and twice the dose of ethanol extract (160 μg/ml) for chloroform extract. The DPPH scavenging activity of *S. wallichii* may be due to the presence of flavonoids and other polyphenols in the extracts as indicated in the present study.

Hydroxyl radicals are highly reactive and are short-lived [49]. They are capable of inducing detrimental effects on the important macromolecules including proteins and nucleic acids. In the Haber-Weiss/Fenton reaction, hydroxyl radicals are generated from hydrogen peroxide in the presence of iron ions [50,51]. The high reactivity of hydroxyl radicals lead to tremendous damage to the cell and its components and subsequently to the organisms as a whole [52]. Therefore, it is very important to remove hydroxyl radicals which cause detrimental effects. The different extracts of *S. wallichii* inhibited the generation of hydroxyl free radicals in a concentration-dependent manner. Kaempferol flavonoid present in *S. wallichii* scavenged OH radicals in an earlier study [45]. Similarly, many plant extracts and flavonoids including mangiferin, and naringin have been found to scavenge hydroxyl free radicals in a concentration-dependent manner [2,21,40,43,48]. Several flavonoids synthesized by different plants as secondary metabolites have been reported to scavenge OH radicals earlier [53,54].

The O_2_^•-^ are generated in biological systems during cellular respiration and as such they are less toxic; however, they are converted into highly reactive OH radical in the presence of iron [55]. Moreover, superoxide anions produced as a result of incomplete metabolism of oxygen damage biomolecules directly or indirectly by forming H_2_O_2_, ^•^OH and peroxynitrite or singlet oxygen [55,56]. Therefore, the removal or neutralization of superoxide radicals is necessary to protect the cells from their deleterious effects. Various extracts of *S. wallichii* inhibited the formation of O_2_^•-^ in a concentration-dependent manner. Kaempferol has been found to scavenge O_2_^•-^ in an earlier report [45]. Other plant extracts and certain plant flavonoids including mangiferin, naringin, quercetin, myricetin and rutin have been found to scavenge superoxide free radical in a concentration-dependent manner [2,21,40,43,48,57].

Nitric oxide is an important cellular signaling molecule involved in many physiological and pathological processes. It is a powerful vasodilator with a short half-life of a few seconds in the blood [55,58]. The nitric oxide radical (NO^•^) is toxic, after reaction with oxygen or superoxide anion radicals. Different extracts of *S. wallichii* reduced the generation of NO^•^ in a concentration-dependent manner. Several plant extracts and plant formulations have been reported to scavenge NO^•^ in a concentration-dependent manner [2,21,40,59]. Similarly, betanin, phyllocactin and betanidin have been reported to scavenge NO radical in a concentration-dependent manner [60]. Kaempferol, myricetin, epigallocatechin gallate, catechin, epicatechin and resveratrol have been reported to scavenge NO radicals [58]. Various flavonoids including delphinidin, pelargonidin, malvin mangiferin and naringin have been found to neutralize NO radicals in earlier studies [40,45,48,54,61–63].

The ABTS^•+^ chromophore is produced through the reaction between ABTS and potassium persulfate which converts ABTS into its radical cation. This radical cation is blue in color and absorbs light at 734 nm [31]. The ABTS^•+^ is reactive towards most antioxidants including phenols, thiols and vitamin C. [64]. The various extracts of *S. wallichii* showed inhibition of ABTS radical production in a concentration-dependent manner. A similar effect has been observed with the extract of *Syzygium cumini*, naringin and mangiferin earlier [40,48,59]. The presence of kaempferol has been reported to scavenge ABTS radicals earlier [45]. FRAP assay had been used to determine antioxidant activity as it is a simple and quick method [65]. The different extracts of *S. wallichii* showed a concentration-dependent rise in FRAP. Several plant extracts have been reported to exhibit antioxidant activity by exhibiting high FRAP values *in vitro* [2,21,42,66]. Likewise, fruits of *Cynometra cauliflora* and *Garcinia atroviridis* have been also reported to possess high FRAP value [67]. Flavonoids from 19 different plants have been found to scavenge ABTS radicals and showed higher FRAP in an earlier study [68].

The exact mechanism of free-radical scavenging by different extracts of *S. wallichii* is not known. However, the phytochemical analysis of *S. wallichii* stem bark has shown the presence of phenols and flavonoids and their concentrations increased with the increase in the amount of extracts. Therefore, the free-radical scavenging and antioxidant activities of *S. wallichii* may be due to the presence of various polyphenols and flavonoids. The presence of kaempferol-3-rhamnoside may have been also responsible for the free-radical scavenging and antioxidant activities of *S. wallichii*.

## Conclusion

The present study demonstrates that all the extracts of *S. wallichii* caused a concentration-dependent inhibition of free radicals and increased  ferric-reducing antioxidant power. These activities of *S. wallichii* may be due to the presence of various phenolic compounds and flavonoids. The ethanol extract showed maximum antioxidant activity followed by the aqueous extract, whereas the chloroform extract showed the least activity. Our study showed that *S. wallichii* possesses antioxidant potential and it might be useful against free radical-induced disorders.

## Future perspective

Inflammation is one of the most important phenomena implicated in various diseases including cardiovascular disorders, diabetes and cancer. The use of antioxidants is helpful in neutralizing free radicals, the main causative factor of inflammatory disorders, and subsequently could be able to prevent free radical-induced ailments. *S. wallichii* use might be helpful in inflammatory disorders and could act as a healthcare aid. However, future studies are required to isolate the active principles. The activity guided isolation of different phytochemicals will be purposeful to establish their antioxidant potential and other disease curing ability in different preclinical models.

**Summary points**

- Free radicals are necessary to carry out various physiological functions in the body; however, their excess production may lead to different health disorders due to triggering of the inflammatory cascade.
- The excess of free radicals may be neutralized by the use of certain exogenous antioxidants.
- Plants synthesize several phytochemicals as secondary metabolites including flavonoids that provide different colors to flowers and fruits and have been consumed by humans since time immemorial.
- *Schima wallichii* a tree belonging to the family Theaceae, which is ethnomedicinally used to treat fever, gonorrhoea, cuts, wounds and lice infection.
- The stem bark powder of *S. wallichii* was extracted in chloroform, ethanol and water and their free-radical scavenging potential was determined.
- The chloroform, ethanol and aqueous extracts of *S. wallichii* scavenged DPPH, hydroxyl, superoxide and nitric oxide radicals in a concentration-dependent manner.
- The chloroform, ethanol and aqueous extracts of *S. wallichii* also showed antioxidant potential as they inhibited the generation of ABTS radical and increased FRAP in a dose-dependent manner.
- The phytochemical analysis of chloroform, ethanol and aqueous extracts of *S. wallichii* showed presence of flavonoids and polyphenols, which increased with increasing concentration.
- The flavonoid contents were maximum at 1000 μg/ml whereas total phenols increased in a concentration-dependent manner up to 900 μg/ml in chloroform, 1000 μg/ml in ethanol and 800 μg/ml in aqueous extracts.
- The free-radical scavenging activities of different extracts may be due to the presence of flavonoids and other polyphenols.
- Our study demonstrates the antioxidant potential of *S. wallichii*, and that its use could be helpful in inhibiting inflammatory health disorders.
